# Life-cycle analysis of greenhouse gas emissions from renewable jet fuel production

**DOI:** 10.1186/s13068-017-0739-7

**Published:** 2017-03-14

**Authors:** Sierk de Jong, Kay Antonissen, Ric Hoefnagels, Laura Lonza, Michael Wang, André Faaij, Martin Junginger

**Affiliations:** 1 Copernicus Institute of Sustainable Development, Utrecht University, Heidelberglaan 2, 3584 CS Utrecht, The Netherlands; 2 European Commission-Directorate for Energy, Transport and Climate, Sustainable Transport Unit, EC-Joint Research Centre, Via E. Fermi 2749, 27027 Ispra, Italy; 3 Systems Assessment Group, Energy Systems Division, Argonne National Laboratory, 9700 S. Cass Avenue, Lemont, IL 60439-4844 USA; 4 Energy Academy Europe, University of Groningen, Nijenborgh 6, Groningen, 9700 AE The Netherlands

**Keywords:** Renewable jet fuel, Aviation, Greenhouse gas emissions, Life-cycle assessment, Alternative jet fuel, Biofuel, Bioenergy, Climate change

## Abstract

**Background:**

The introduction of renewable jet fuel (RJF) is considered an important emission mitigation measure for the aviation industry. This study compares the well-to-wake (WtWa) greenhouse gas (GHG) emission performance of multiple RJF conversion pathways and explores the impact of different co-product allocation methods. The insights obtained in this study are of particular importance if RJF is included as an emission mitigation instrument in the global Carbon Offsetting and Reduction Scheme for International Aviation (CORSIA).

**Results:**

Fischer–Tropsch pathways yield the highest GHG emission reduction compared to fossil jet fuel (86–104%) of the pathways in scope, followed by Hydrothermal Liquefaction (77–80%) and sugarcane- (71–75%) and corn stover-based Alcohol-to-Jet (60–75%). Feedstock cultivation, hydrogen and conversion inputs were shown to be major contributors to the overall WtWa GHG emission performance. The choice of allocation method mainly affects pathways yielding high shares of co-products or producing co-products which effectively displace carbon intensive products (e.g., electricity).

**Conclusions:**

Renewable jet fuel can contribute to significant reduction of aviation-related GHG emissions, provided the right feedstock and conversion technology are used. The GHG emission performance of RJF may be further improved by using sustainable hydrogen sources or applying carbon capture and storage. Based on the character and impact of different co-product allocation methods, we recommend using energy and economic allocation (for non-energy co-products) at a global level, as it leverages the universal character of energy allocation while adequately valuing non-energy co-products.

**Electronic supplementary material:**

The online version of this article (doi:10.1186/s13068-017-0739-7) contains supplementary material, which is available to authorized users.

## Background

The aviation industry emits roughly 2% of global anthropogenic greenhouse gas (GHG) emissions [1]. Despite a projected fourfold increase in CO_2_ emissions in 2050 relative to 2010 [2], aviation was excluded from the recent COP21 Paris Agreement [3]. The International Air Transport Association (IATA) has set an industry target to achieve carbon neutral growth after 2020 and reduce emissions by 50% in 2050 (referenced to 2005). Besides efficiency improvements in technology and operations, the adoption of renewable jet fuel (RJF), a Jet A-1 substitute derived from biomass, is expected to make an important contribution [4]. The International Civil Aviation Organisation (ICAO) recently agreed to develop a Global Market-based Measure (GMBM) to achieve carbon neutral growth after 2020 [5]. In this scheme, aircraft operators should offset any annual increase in the GHG emissions beyond 2020 from international aviation between participating states using the Carbon Offsetting and Reduction Scheme for International Aviation (CORSIA). The scheme is currently approved until 2035. Consumption of RJFs may also be included as part of a basket of measures [5].

The contribution of RJF to the emission reduction ambitions in aviation depends on the market penetration of RJF and its GHG emission reduction potential. To date, the market penetration of RJF has been negligible because of high prices and limited production capacity. Prior studies have explored the techno-economic feasibility and technology readiness of different RJF conversion pathways [6–11]. A positive GHG emission balance and sustainability impact (e.g., on water use, land use, biodiversity, etc.) is essential for RJF to contribute to a more sustainable aviation industry.

Various GHG emission performance assessments have been conducted for road biofuels, including comparisons between different conversion pathways [12–15]. Previous studies have shown the GHG emission performance is impacted significantly by methodological choices (especially allocation methods for co-products) and spatiotemporal variability in input data (e.g., feedstock yields or electricity mix) [16–21]. Although RJF can be produced from similar feedstocks as road biofuels,^1^ conversion and downstream handling may deviate due to different fuel specifications and higher quality standards. These standards generally require more stringent upgrading, thus affecting yields and/or hydrogen consumption. Moreover, a thorough understanding of the impact of different methodological frameworks on the GHG emission performance of RJF is necessary, because the use of RJF in a global carbon offsetting scheme requires a global methodological meta-standard.

Prior analyses have considered the GHG emission performance of several RJF conversion pathways [21–28]. A comparison of the results is challenging due to diverging methodologies and input data. This study expands the comparative base by examining the GHG emission performance of six RJF conversion technologies: Hydroprocessed Esters and Fatty Acids (HEFA), Fischer–Tropsch (FT), Hydrothermal Liquefaction (HTL), pyrolysis, Alcohol-to-Jet (ATJ) and Direct Sugars to Hydrocarbons (DSHC; also commonly referred to as Synthetic Iso-paraffinic fuel, SIP). Additionally, this analysis shows the impact of different co-product allocation methods. As such, the objectives of this study are to (1) compare the GHG emission performance of RJF conversion pathways using different allocation procedures, (2) discuss potential improvements of the GHG emission performance of RJF, and (3) provide input for the development of a methodological meta-standard for the calculation of the GHG emission performance of RJF.

## Methods

### LCA framework

A life-cycle analysis (LCA) framework can be used to assess the environmental impact across the entire product life-cycle. Methodology and default values are often standardized within a certain regulatory context, such as the EU Renewable Energy Directive (RED) and US Renewable Fuel Standard (RFS). A number of standardized approaches and respective calculation tools exist, of which prominent ones include the Greenhouse gasses, Regulated Emissions and Energy use in Transportation (GREET), BioGrace, and GHGenius (used in the US, EU and Canada, respectively). This study utilized the GREET model (GREET.net v1.3.0.12844, database version 12384), as it already included some RJF conversion pathways [24, 29, 30]. Furthermore, it gives the opportunity to compare and add pathways in a comprehensive yet transparent way. Default values for the reference year 2020 were used to assess the short-term GHG emission performance of RJF conversion pathways.

### Functional unit

The conversion pathways were compared on the basis of their GHG emissions in gCO_2eq_ per MJ of RJF. The GHG emissions considered were CO_2_, CH_4_ and N_2_O using their 100-year global warming potential (1, 25 and 298, respectively), in line with the United Nations Framework Convention on Climate Change reporting guidelines [24, 31].

### System boundaries

**Fig. 1:**
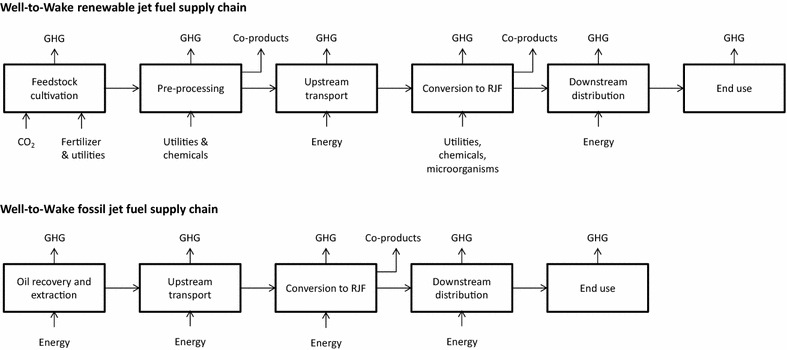
A schematic overview of the RJF supply chain and the system boundaries used in this study

The assessment covered well-to-wake (WtWa) GHG emissions, expressed as CO_2eq_, including emissions from feedstock cultivation and pre-processing, upstream logistics, conversion to RJF, downstream distribution, and end use (Fig. 1). Upstream transport comprises the transport from the feedstock production site or pre-processing facility to the conversion facility. Downstream distribution includes the transportation of the RJF to a blending terminal, blending operations, transportation to the airport tank farm and storage. Non-CO_2_ emissions from jet fuel combustion were excluded from the analysis, as reported combustion data were only found for HEFA and FT RJF. Furthermore, as the chemical properties of RJF are by definition closely related to fossil jet fuel, it was assumed that there is no significant difference in GHG emissions from combustion, as was demonstrated for HEFA and FT RJF [24, 32–35]. CO_2_ emissions from the combustion of RJF are treated to be zero under the assumption of carbon neutrality [18].

### Land use change

Emissions from direct and indirect land use change (LUC) can have a large impact on the GHG emission performance of conversion pathways [12, 22]. Emissions from direct LUC are caused by changes to the above- and below-ground carbon stocks as a result of changing former land use to cultivate biomass for bioenergy purposes. Changing land use for biomass cultivation or diverting existing feedstock flows for bioenergy purposes may instigate land use changes elsewhere to restore initial production level of food, feed and materials, causing indirect LUC emissions. The larger part of LUC emissions typically occur at the start of a project; as such, its impact can be affected significantly by the method used to amortize emissions over a given time period [36].^2^

Depending on the context, LUC-related GHG emissions may be positive (net emissions) or negative (net sequestration). Negative LUC-emissions may occur for highly productive feedstocks with a low fertilizer requirement (e.g., perennial grasses) which sequester more above- and below-ground carbon than the reference vegetation, especially when grown on degraded or marginal lands (which mitigates indirect LUC effects as well) [37–39]. Conversely, conversion of large carbon stores (e.g., (tropical) forests, peatlands or prairie) into high-input croplands (e.g., palm oil or corn) may lead to high positive LUC emissions. Although important, these impacts are challenging to quantify, surrounded by considerable uncertainties and highly dependent on context-specific circumstances such as soil type, previous land use and management practices (please see Wicke et al. [40] for a comprehensive review of LUC-related GHG emissions from biofuels) [40–43]. Moreover, quantification of these effects should be considered in a broader context; for example, agricultural zoning, improved management or intensification measures in agriculture may mitigate the indirect LUC GHG emissions from bioenergy [40, 44]. As this analysis focused on the performance of the conversion pathway, LUC emissions were excluded from this analysis.

### Conversion pathway scope

**Fig. 2:**
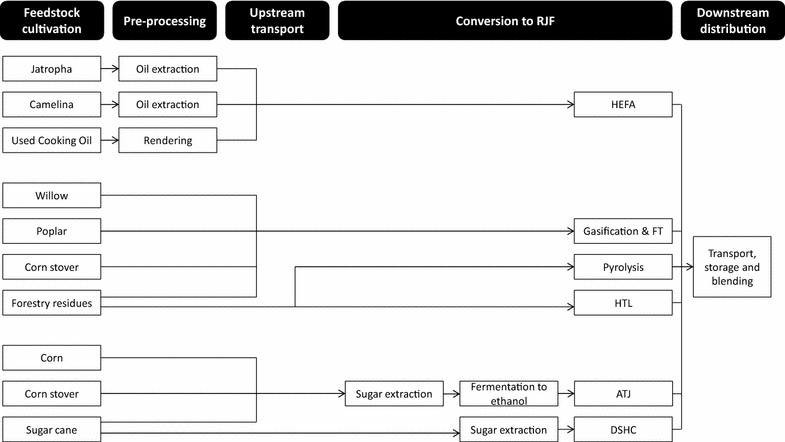
The scope of conversion pathways

The scope included technologies which are or are expected to become commercially available in the near-term, namely Hydroprocessed Esters and Fatty Acids (HEFA), Fischer–Tropsch (FT), Hydrothermal Liquefaction (HTL), pyrolysis, Alcohol-to-Jet (ATJ) and Direct Sugars to Hydrocarbons (DSHC), see Fig. 2.^3^ The selected feedstocks include sugar/starch (sugarcane and corn), lignocellulosic (poplar, willow, corn stover and forestry residues), and oil feedstocks (used cooking oil, jatropha and camelina), as these feedstocks are currently used or have been considered for RJF production (this is, however, not an exhaustive list).

### Methods to deal with co-products

The co-product allocation method in an LCA can have a profound impact on the GHG emission performance of a product [18, 20–22, 25], especially when the yield of co-products is high compared to the main product (see also Additional file 1). GHG emissions can be allocated to the co-products according to their energy, mass and economic value [18, 20, 45, 46]. Alternatively, the displacement method (or system expansion) awards an emission credit to co-products based on the yield of the co-product and the GHG emission intensity of the displaced product (e.g., the fossil counterpart of the co-product). While energy allocation yields strictly positive emission intensities (except bioenergy pathways with carbon capture and storage), the displacement method may give negative emission intensities in case the emission credits exceed the total system emissions.

Benefits and drawbacks exist for each method; the suitability of a particular method largely depends on the production system and the co-products. The International Standards Organisation (ISO) [46] deems the use of the displacement method most appropriate as it represent the potential GHG emission mitigation effects of producing co-products. However, it requires additional system choices regarding the displaced product and the associated avoided emissions [20]. Furthermore, when the yield of the co-products is significant compared to the main product, the choice of main product and co-product can have a decisive impact on the results [20, 47]. Allocation methods are indifferent to the choice of main product. Moreover, mass and energy allocation are based on physical properties of the co-product and are thus universally applicable and less susceptible to methodological choices and uncertainties. This is a key motivation for regulators, including the European Union, to adopt this method in a normative context [20]. However, mass allocation can only be applied to co-products having a mass and is hence unsuitable for systems producing immaterial products such as electricity. Energy allocation may not rightfully capture the value of non-energy co-products. For example, camelina meal, which can be used as an animal feed, is allocated more emissions when it is valued for its nutritional value (substituting soybean meal or corn) than when it is valued for its energy content [25]. The last method, economic allocation, captures the economic value of the co-product. However, a price ratio may be challenging to establish for novel non-commoditized products and could be highly affected by price fluctuations, geographical location or market distortions (e.g., monopolies or subsidies) [20, 45]. As such, economic allocation is earmarked by the International Standards Organisation (ISO) as a last-resort methodological option, when other methods prove inapt [45, 46].

In this study both energy allocation and the displacement method were used for non-energy co-products as they are employed in two prominent regulatory frameworks, the EU Renewable Energy Directive and the US Renewable Fuel Standard, respectively (Table 1) [48, 49]. Energy allocation was used for all fuel co-products (i.e. diesel, gasoline, heavy fuel oil, propane, methane and naphtha), as this is common practice for (sub-)processes which produce mainly fuels, because it captures the energy value of the product and is relatively indifferent to the choice of co-product and variations in product slate [21, 50, 51]. Hence, essentially, two analyses were conducted; one using solely energy allocation and one using a hybrid method, integrating the displacement method and energy allocation. An analysis using only the displacement method was not conducted, as such analysis was shown to be very sensitive to the choice of main product, especially if the yield fraction of the main product is low compared to other fuel co-products produced by the same system. [21].

**Table 1:** An overview of biofuel regulation in the EU renewable energy directive and US renewable fuel standard

	EU renewable energy directive^a^	US renewable fuel standard	
Co-production allocation method for non-fossil products	Energy allocation except for cogeneration of heat and (excess) power	Displacement method	
GHG reduction threshold (compared to the fossil fuel baseline)	35% for all biofuels 50% from 1 January 2017 for all existing installations 60% from 1 Jan 2018 for installations commencing production after 5 October 2015		
		*Biofuel category*:	*Applicable to*:
		Cellulosic biofuel: 60%	Lignocellulosic feedstocks
		Advanced biofuel: 50%	All feedstock except corn starch
		Biomass-based diesel: 50%	Oil feedstocks
		Renewable fuels (conventional biofuels): 20%	Typically refers to corn ethanol
Fossil fuel baseline	83.8 g CO_2eq_/MJ	Diesel type fuels: 91.8 CO_2eq_/MJ Gasoline type fuels: 93.3 g CO_2eq_/MJ	

^a^In 2015 the EU introduced a 7% cap on biofuels from food crops grown on agricultural land and an indicative 0.5% target for advanced biofuels to reduce the risk of indirect LUC effects

### Fossil baseline

The results were compared to the baseline WtWa emissions of fossil jet fuel. Considerable ranges for jet fuel exist depending on crude oil quality and processing technique; for the US a range between 80.7 and 109.3 g CO_2eq_/MJ was reported [52], while for the EU a range of 80.4-105.7 was found [53].^4^ The average WTWa emission intensity for conventional jet fuel (87.5 g CO_2eq_/MJ) consumed in the US was used as the fossil baseline such that it matches the geographical scope of the input data [52]. This baseline is in between the fossil fuel baselines for transport fuels used in EU and US regulatory frameworks (Table 1).

### Threshold values

The EU and US regulatory frameworks use GHG emission reduction threshold values to define which biofuels are eligible to count towards renewable fuels targets (Table 1). These thresholds originate from policy objectives (e.g. emissions reduction, sustainability requirements, security of supply) rather than being of technical origin. Whereas the EU renewable energy directive has increasingly higher reduction thresholds over time, the US renewable fuel standard has separate reduction thresholds for different categories of biofuels which are fixed in time. The biofuel categories in the US renewable fuel standard are based on the feedstock-technology combination.

The results of this study were compared to the GHG emission reduction threshold as specified for biofuels in the EU renewable energy directive and US renewable fuel standard to provide an indication of the eligibility of the RJF conversion pathways under both regulatory schemes. It is an order-of-magnitude screening only, primarily because this assessment does not include sustainability indicators acting as exclusion criteria and LUC emissions. Also, it uses (slightly) different fossil baselines, default values and assessment methodologies, especially relative to the EU regulatory framework.

## Life cycle inventory

This section discusses the system configurations and most important assumptions used in this study. A full overview of the input data can be found in Additional file 2.

### Geographical origin of the data

Input values may vary across different world regions due to e.g., farming practice, feedstock yield or process design. In this study, RJF was assumed to be consumed in the US. Most feedstock cultivation and RJF production was situated in the US, except for sugarcane-based DSHC and ATJ for which feedstock cultivation and conversion to RJF occurs in Brazil. In these cases, transportation of the RJF to and distribution in the US was added for consistency. Default values in GREET were used where available. The life-cycle inventory was complemented with data from recent studies for those feedstocks and technologies not available in GREET. Energy use for blending and storage was obtained from BioGrace [54], but US emission factors were used to calculate the associated emissions.

### Conversion pathway description

This study comprises six conversion technologies:

- *Hydroprocessed esters and fatty acids (HEFA)* The HEFA technology uses hydrogen to deoxygenate and saturate the fatty acid carbon chains. Carbon chains are sized to fit the diesel and jet range using selective hydrocracking and/or isomerization. The values used in this study were taken from the GREET database, which is based on the UOP Ecofining process [24, 29, 47, 52].
- *Gasification and Fischer–Tropsch (FT)* Lignocellulosic biomass is gasified to produce syngas. The syngas is converted to RJF, diesel, gasoline, propane and methane through FT synthesis. Electricity is generated from excess steam from gasification and FT synthesis. Process performance data were taken from Swanson et al. [55]. As the reference study did not consider RJF production, it was assumed that the diesel output could be split in 25% RJF-ranged hydrocarbons and 75% diesel-ranged hydrocarbons. No additional emissions were taken into account as distillation was already considered in the process design.
- *Pyrolysis* The pyrolysis process design was adopted from Tews et al. [56]. In the process, feedstocks are dried (using waste heat from char combustion), ground (using electricity) and consequently converted at elevated temperatures (~500 °C) to bio-oil, gas and char [57]. The bio-oil is consequently converted to a mixture of hydrocarbons by hydrodeoxygenation. Char is combusted to produce steam. Again, a 25–75% RJF-diesel split was applied to the diesel output.
- *Hydrothermal liquefaction (HTL)* The HTL process design was also based on Tews et al. [56]. The HTL process converts wet feedstocks (no drying required) into a biocrude using water as a medium. Compared to pyrolysis it is operated at more modest temperatures (250–550 °C), but elevated pressures (5–25 MPa) [58]. As the HTL biocrude contains less oxygen than the pyrolysis bio-oil, the hydrodeoxygenation step requires less hydrogen. Again, a 25–75% RJF-diesel split was applied to the diesel output.
- *Alcohol to jet (ATJ)* The ATJ platform converts alcohols (e.g. ethanol, butanol) to hydrocarbons. In this study, we use the ATJ pathway available in the GREET excel model. This pathway upgrades ethanol to RJF, diesel and naphtha through dehydration, oligomerization and hydroprocessing [26, 30]. Data for ethanol production through fermentation of sugarcane, corn (including milling processes) and corn stover were adopted from GREET [29].
- *Direct sugars to hydrocarbons (DSHC)* In the DSHC process, sugars are fermented to farnesene, a branched C-15 molecule with four double bonds. The double bonds are saturated using hydrogen to produce farnesane. We used data for the DSHC process including the sugarcane milling from Klein-Marcuschamer et al. and Cox et al. which are based on the Amyris process [8, 27]. Unlike these studies, we assume both sugar and molasses were used to produce biofuels. Although farnesane is eligible for 10% blending with fossil jet fuel, Klein-Marcuschamer et al. process design includes additional hydrocracking and hydroisomerization, which produces an enhanced RJF with a higher blend level but also increases the hydrogen consumption. Both the ‘increased blend level’ and ‘10% blend level’ cases were considered here. The former case is based on the hydrogen consumption as specified in Klein-Marcuschamer et al. The hydrogen consumption for the latter case was approximated by taking 120% of the stoichiometric hydrogen required for farnesene saturation. In this case, it was assumed that the farnesane is used as RJF only; no co-products were produced.

The process performance indicators of the RJF conversion technologies are listed in Table 2 and Additional file 2. The reader is referred to Mawhood et al. [10] for a more elaborated description of the conversion technologies and their respective CAAFI fuel readiness level.^5^

**Table 2:** Key process assumptions regarding the RJF conversion technologies [8, 29, 30, 55, 56]

Process	Unit	HEFA [29]	FT [55]	Pyrolysis [56]		HTL [56]		ATJ [30]	DSHC [8]	
Sub-process				Ex situ	In situ	Ex situ	In situ		Increased blend level	10% blend level
Inputs										
Feed	MJ feed/MJ RJF	1.17	12.93	26.39	26.39	16.89	16.89	1.49^a^	6.28	3.25
Natural gas consumption^b^	MJ/MJ RJF	0.18								
Electricity consumption	MJ/MJ RJF	0.005			1.53	0.21	0.86	0.03		
Hydrogen consumption	MJ/MJ RJF	0.15		5.44		1.31		0.08	0.52	0.12
Hydrogen feedstock		Natural gas		Natural gas	Process off-gases	Natural gas	Process off-gases and waste water	Natural gas	Natural gas	Natural gas
Outputs										
Co-product allocation ratio										
RJF	Normalized	1	1	1	1	1	1	1	1	1
Diesel	MJ/MJ RJF		3.00	2.95	2.95	2.95	2.95	0.12	0.15	
Gasoline	MJ/MJ RJF		1.69	7.88	7.88	4.57	4.57			
Heavy fuel oil	MJ/MJ RJF			2.17	2.17	1.65	1.65			
Naphtha	MJ/MJ RJF	0.14						0.21	0.54	
Propane	MJ/MJ RJF	0.10	0.49							
Methane	MJ/MJ RJF		0.24							
Electricity	MJ/MJ RJF		0.45	0.51					0.13	0.07

^a^Feedstock is ethanol

^b^Excluding natural gas used for hydrogen generation

### Hydrogen generation

All pathways require hydrogen except FT, HTL (in situ) and pyrolysis (in situ). In the base case it was assumed that hydrogen was produced through steam methane reforming (SMR) of natural gas, which corresponds to the current production practice of hydrogen. For pyrolysis and HTL, ex situ (SMR of natural gas) and in situ hydrogen production were considered. In the pyrolysis in situ case, hydrogen was produced through SMR of process off-gases; in the HTL in situ case hydrogen was produced through SMR of off-gases from the process and anaerobic digestion of the waste water. Ex situ hydrogen consumption was calculated from mass and energy balances presented in Tews et al. [56]. The feeds used for hydrogen generation in the in situ case were utilized to power the process in the ex situ case, hence explaining the lower electricity consumption in the ex situ case.

### Allocation and displacement ratios

All conversion pathways produce non-fuel and/or fuel co-products. Table 2 shows the co-product allocation ratios for fuel co-products. Table 3 provides an overview of the co-product allocation ratios applied for non-energy co-products (a subgroup of non-fuel co-products) and electricity (considered an energy and non-fuel co-product) for both the energy allocation and displacement method.

**Table 3:** Allocation ratios for non-energy co-products and electricity [25, 29, 30, 56, 59–63]

Supply chain component	Applicable for pathway	Main product	Co-product	Co-product allocation ratio r_A_ Co-product/main product		Displaced product	Displacement ratio r_D_	Emission factor	Reference
				MJ/MJ	g/MJ		g displaced product/g co-product	g CO_2eq_/g displaced product	
Non-energy co-products									
Camelina oil extraction	HEFA	Camelina oil	Camelina meal	0.64	47.79	Soybean meal	0.77^a^	0.53	[25, 59, 60]
Corn dry mill ethanol production w/o corn oil extraction^b^	ATJ	Ethanol	Distillers grain solubles	0.68	31.74	Corn	0.78	0.29	[29]
						Soybean meal	0.31	0.53	
						Urea	0.02	1.22	
Corn dry mill ethanol production w/ corn oil extraction^b^	ATJ	Ethanol	Distillers grain solubles	0.65	30.36	Corn	0.78	0.29	[29]
						Soybean meal	0.31	0.53	
						Urea	0.02	1.22	
		Ethanol	Corn oil	0.04	1.06	Soy oil	1.00	0.53	[29]
Corn wet mill ethanol production^b^	ATJ	Ethanol	Corn gluten meal	0.15	6.87	Corn	1.53	0.29	[29]
						Urea	0.02	1.22	
		Ethanol	Corn gluten feed	0.56	29.74	Corn	1.00	0.29	[29]
						Urea	0.02	1.22	
		Ethanol	Corn oil	0.21	5.52	Soy oil	1.00	0.53	[29]

Supply chain component	Applicable for pathway	Main product	Co-product	Co-product allocation ratio r_A_ Co-product/main product		Displaced product	Displacement ratio r_D_	Emission factor	Reference
				MJ/MJ			MJ displaced product/MJ co-product	g CO_2eq_/MJ displaced product^c^	
Electricity (co-product)									
FT synthesis	FT	RJF	Electricity	0.45		US grid electricity	1.00	137.88	[30]
Jatropha oil extraction	HEFA	Jatropha oil	Electricity^d^	0.34		US grid electricity	1.00	137.88	[29]
Pyrolysis	Pyrolysis ex situ case	RJF	Electricity	0.51		US grid electricity	1.00	137.88	[54]
Ethanol from corn stover	ATJ	Ethanol	Electricity	0.10		US grid electricity	1.00	137.88	[61]
Ethanol from sugarcane	ATJ	Ethanol	Electricity	0.22		Brazilian grid electricity	1.00	26.52	[62]
Sugarcane milling	DSHC (increased blend level)	RJF	Electricity^e^	0.13		Brazilian grid electricity	1.00	26.52	[63]
Sugarcane milling	DSHC (10% blend level)	RJF	Electricity^e^	0.07		Brazilian grid electricity	1.00	26.52	[63]

^a^Based on the ratio between the average protein content of camelina (36.2%) and soybean meal (47%)

^b^GREET uses a weighted average of three different corn ethanol technologies. Dry mill ethanol production without corn oil extraction, dry mill ethanol production with corn oil extraction, and wet mill ethanol production respectively produce 18.23% , 72.91% and 8.87% of the total produced ethanol

^c^For electricity production, an average emission factor without transmission and distribution losses was used. For electricity consumption, these losses were included. For pathways located in Brazil, a much lower emission factor was used due to the high diffusion of hydropower in the electricity mix

^d^From the combustion of jatropha husks, shells and meal

^e^From the combustion of bagasse, after deduction of internal use of heat and power in the DSHC process

## Results

### Comparison between pathways

**Fig. 3:**
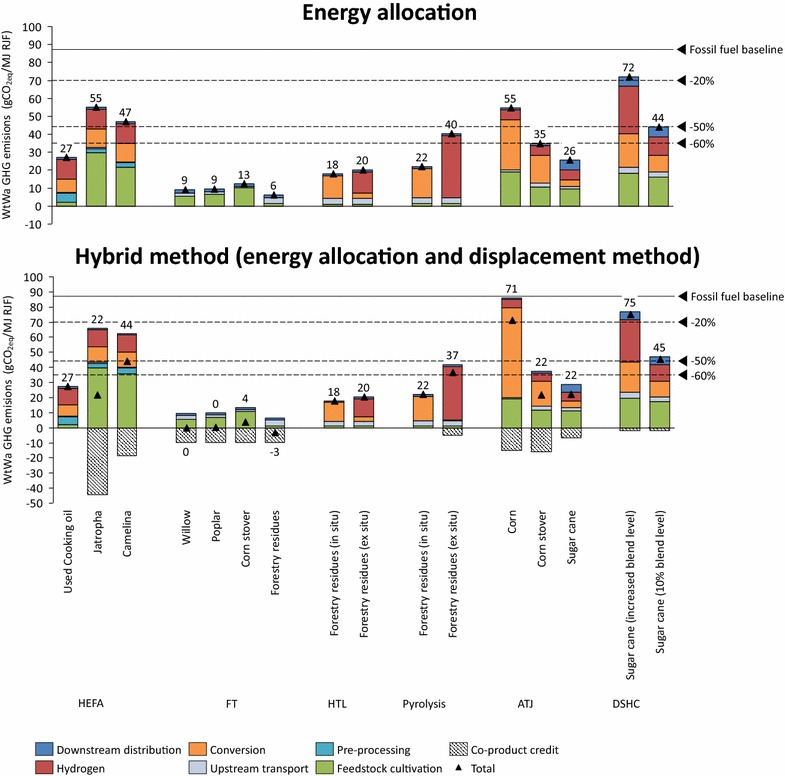
WtWa GHG emission performance of RJF conversion pathways

Figure 3 shows the WtWa GHG emissions per conversion pathway for energy allocation and the hybrid method. FT yields consistently low WtWa GHG emissions across all feedstocks and both allocation methods, mainly due to the self-sufficiency of the process and excess electricity production. Corn-based ATJ and sugarcane-based DSHC (increased blend level case) show the highest WtWa GHG emissions in both methods. For corn-based ATJ this is caused by high fossil energy use during ethanol production and high emissions from fertilizer use. For DSHC, the low conversion yield and high hydrogen consumption are the main contributors to a high GHG footprint. Jatropha and camelina-based HEFA also show particularly high cultivation emissions. While per-hectare use of fertilizer and other inputs could be small for jatropha and camelina, the oil yield is usually low, leading to high emissions per unit of oil. In almost all processes hydrogen is an important contributor to the overall WtWa GHG emissions. In situ hydrogen production generally yields lower WtWa GHG emissions than ex situ hydrogen production; the emissions avoided by producing hydrogen from off-gas instead of natural gas offset the emissions related to increased electricity use (valid for the US electricity mix). The benefits of in situ production are stronger for the pyrolysis process as the upgrading of pyrolysis oil requires large amounts of hydrogen and the process off-gas already contains high concentrations of hydrogen. For RJF conversion pathways situated in Brazil (sugarcane-based pathways), the emissions from downstream distribution increase slightly due to international transport while emissions from electricity use (or co-product credit) are reduced. This reduction is because Brazil’s average electricity mix has a lower emission factor compared to the US, particularly due to a high share of hydropower.

Most pathways yield GHG emissions reductions exceeding 60% compared to fossil jet fuel and can therefore comply with the most stringent emission reduction thresholds of the EU renewable energy directive and US renewable fuel standard. Whereas DSHC (increased blend level) is above or close to the lowest thresholds for biofuels irrespective of allocation method, the performance of jatropha-based HEFA or corn-based ATJ highly depends on the allocation method used. It is worth reminding that this assessment does not include LUC emissions, and therefore could over- or underestimate the GHG emission performance of these conversion pathways for a specific context.

Residues and lignocellulosic crops generally show better emission mitigation potential than food crops, because of low emissions related to fertilizer use, feedstock cultivation or feedstock collection. RJF produced from highly productive food crops in combination with an efficient conversion process (i.e., sugarcane-based ATJ) is also able to meet the strictest GHG emission reduction thresholds currently applied.

### Comparison between allocation methods

Figure 3 shows that the allocation method applied has a significant effect on the GHG emission performance for some pathways. As described in Additional file 1, the difference between energy allocation and the hybrid method increases for conversion pathways producing large amounts of co-products. Moreover, the hybrid method tends to yield lower WtWa GHG emissions for conversion pathways producing co-products which displace products whose emission intensity exceeds the emission intensity of the system (before allocation).

Particular differences are observed for FT and jatropha-based HEFA. Although the co-product (electricity) is valued for its energy content in both methods, they still yield disparate results. In these cases, the emission intensity of the displaced product (grid electricity) far exceeds the emission intensity of the system, hence leading to the hybrid method yielding lower GHG emission results than energy allocation. Similar dynamics are at the origin of the lower emission intensity of pyrolysis (ex situ) and corn stover-based ATJ for the hybrid method. On the contrary, sugarcane-based DSHC yields higher emissions using the hybrid method because of the low emission intensity of Brazilian electricity combined with a high GHG emission profile of the conversion pathway. Despite a relatively high co-product allocation ratio for camelina-based HEFA, the moderate displacement ratio and low emission intensity of soy meal yields only a small decrease in WtWa GHG emissions for the hybrid method. This pathway will be examined more closely in the sensitivity analysis.

Conversely, corn ATJ shows higher emissions using the hybrid method. This is to be ascribed to its co-products (distillers grain solubles, corn oil, corn gluten meal and corn gluten feed) displacing products with low emission intensities relative to the total system, which makes energy allocation more attractive than the displacement method.

Two out of sixteen pathways change threshold category after applying a different allocation method. Whereas jatropha-based HEFA meets a lower threshold category using the hybrid method, corn-based ATJ is demoted one category.

## Sensitivity analysis

### Alternative allocation methods for non-energy co-products

To illustrate the impact of different allocation methods we apply mass and economic allocation to the camelina-based HEFA pathway in which large amounts of camelina meal are produced. Similar to the base results, energy allocation was used for the remaining fuel co-products (i.e., propane and naphtha). For mass-based allocation, we used an allocation ratio of 1.78 kg camelina meal/kg camelina oil. For economic allocation, the ratio between soy oil and soy meal was used as a proxy to determine the allocation ratio, as price data for camelina meal and oil were not available. A price for camelina meal and oil was derived from this ratio using a displacement ratio of 0.77 kg camelina meal/kg soy meal and 1 kg camelina oil/kg soy oil, respectively. A mean, minimum and maximum (0.34, 0.29 and 0.45 $/kg camelina meal per $/kg camelina oil) allocation ratio was found, based on a 10-year series of monthly price ratios between soy oil and soy meal [64].

**Fig. 4:**
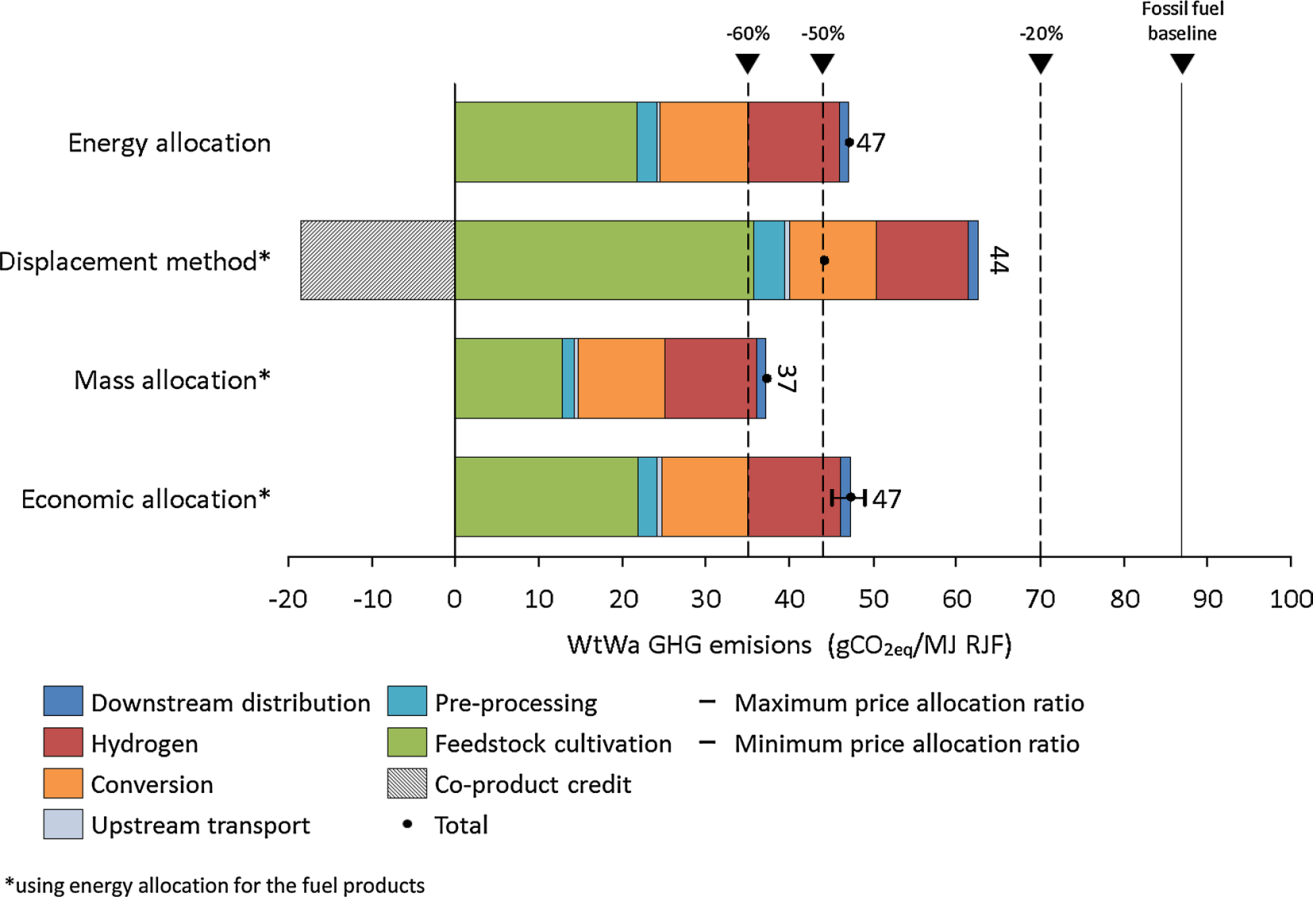
WtWa GHG emissions for the HEFA camelina pathway using different co-product allocation methods for camelina meal

Figure 4 shows that the WtWa GHG emissions for the camelina-based HEFA pathway range between 37 and 49 g CO_2eq_/MJ RJF for different allocation methods. Whereas energy allocation assigns a relatively small share of emissions to the meal, mass allocation allocates a high share of emissions to the meal due to the large mass of meal produced. Economic allocation shows a modest range of ±5% due to variability of price ratios. Although the displacement method is shown as a point value here, different assumptions regarding displacement ratio, displaced product or emission intensity of the displaced product may change the result substantially, as was shown in other studies for, e.g., camelina and jatropha-based HEFA RJF [21, 22, 25].

### Yield, fertilizer use and hydrogen use

In Fig. 3, feedstock cultivation, hydrogen use and conversion were shown to have an important contribution to the overall WtWa GHG emissions. Therefore, a sensitivity analysis was performed to determine the impact of the hydrogen, N fertilizer and conversion yields. Ranges for conversion yields were adopted from a survey of technology performance data (see Additional file 3) [6]. Ranges in hydrogen emissions originate from variability in hydrogen consumption or emission intensity of hydrogen production. Emissions from N fertilizer input may vary for different management practices, cultivation locations or calculation methods (see Additional file 2). Both parameters were varied by ±20% to illustrate the sensitivity of the WtWa GHG emissions to variance in these parameters. The ranges were inserted as single permutations and simultaneous permutations (as indicated by ‘All’). The results were calculated using energy allocation.

**Fig. 5:**
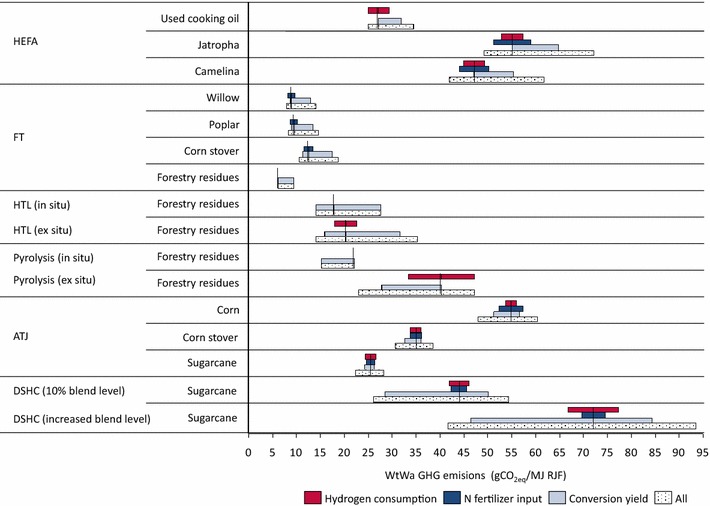
Sensitivity analysis on hydrogen consumption, N fertilizer input and conversion yield (energy allocation)

Figure 5 shows that the general merit order is retained in the sensitivity analysis. Whereas the majority of the pathways show modest ranges (<±20% for simultaneous permutations), pyrolysis (ex situ) and DSHC (high blend level) show relatively large ranges, mainly due to hydrogen being an important determinant for the performance of these conversion pathways and the uncertainty regarding the conversion yield. Fertilizer input is shown to have a minor impact on the results. Furthermore, it is shown that the Base case considers relatively pessimistic yields for DSHC and pyrolysis, while being optimistic for HEFA, FT and HTL.

### Hydrogen production method

The base results assume hydrogen production using SMR of natural gas. Technological advancements and a higher penetration of renewable electricity can make more sustainable hydrogen generation processes technically and economically feasible. Two other processes were assessed to show the impact of such developments: (1) electrolysis using renewable electricity from wind, solar and biogenic waste and (2) gasification of biomass (switchgrass was taken as a proxy for biomass). These pathways were adopted from GREET [29]. The results were calculated using the energy allocation method.

**Fig. 6:**
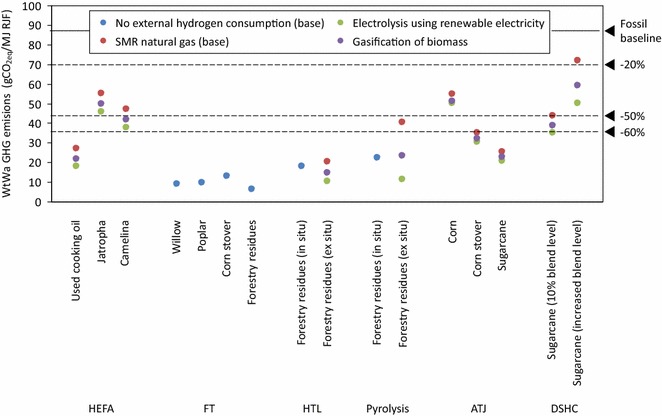
Sensitivity analysis on the hydrogen source (energy allocation)

Figure 6 illustrates that alternative hydrogen generation methods can reduce the WtWa GHG emissions significantly and shift the merit order, especially for pathways for which hydrogen consumption is responsible for a high share of the total emissions such as ex situ pyrolysis (−71%), ex situ HTL (−48%), DSHC (−20 to 30%) and UCO-based HEFA (−34%). For electrolysis, the majority of the conversion pathways show WtWa GHG emissions below the 50% emission reduction threshold.

## Discussion

### Conversion pathway WtWa GHG emission performance

The first aim of this study was to compare the GHG emission performance of RJF conversion pathways using different allocation procedures. In terms of feedstock, it is shown that residues or lignocellulosic crops yield low WtWa GHG emissions, irrespective of conversion pathway or allocation method. The food and oil crops under consideration were generally characterized by higher feedstock cultivation emissions, which originated particularly from the high fertilizer use (except for sugarcane). In terms of technology, hydrogen consumption and conversion yield were found to be important determinants of GHG emission performance. Upstream transport and downstream distribution only contributed marginally to the overall WtWa GHG emissions. Although considerable uncertainty exists, the merit order of the pathways considered is quite robust to changes in key input parameters. Wide ranges were particularly observed for DSHC (high blend level) and pyrolysis (ex situ) due to uncertainty regarding the conversion yield.

It is stressed that the results were obtained for a specific spatiotemporal context. The spatial component may influence emissions from feedstock cultivation and the carbon intensity of utilities and fossil jet fuel. [19, 52, 53, 65, 66] Improvements inside and outside the production system may positively affect the GHG emission performance of RJF over time (see “Improving the GHG emission performance of RJF production” section).

Table 4 shows a comparison of study results with existing studies using energy allocation, the displacement method, or a hybrid method. The ranges found are largely ascribed to variability in methodological approach (e.g., system boundaries or life cycle inventory elements, i.e., some include land use change emissions) or input data (e.g., co-product allocation ratios, conversion yields). Despite this variability, studies seem to agree on the superior WtWa GHG emission performance of FT RJF, irrespective of the feedstock or allocation method used. Greater methodological variability in the application of the displacement method causes wider ranges in GHG emission performance. The observed difference between results from both allocation methods concurs with existing literature, particularly for conversion pathways with high co-product allocation ratios or co-products which effectively displace emission-intensive products (e.g., electricity) [20, 21, 25, 67].

**Table 4:** A comparison of study results with existing literature [21, 22, 24–28, 52, 56, 68–70]

Technology^a^	Feedstock	Energy allocation		Reference	Displacement method		Reference
		This study	Prior studies		This study	Prior studies	
		g CO_2eq _/MJ	g CO_2eq_/MJ		g CO_2eq_/MJ	g CO_2eq_/MJ	
HEFA	UCO	28	17–21	[68]	28	–	
	Jatropha	55	37–55	[21, 22, 28]	21	−134 to 63	[21, 22, 52]
	Camelina	47	18–47	[25, 28]	44	−17 to 60	[25, 69]
FT	Willow	9	–		−7	−17 to 10	[24, 70]
	Poplar	10	–		−6	−17 to 10	[24, 70]
	Corn Stover	13	8–11	[28]	−3	9 to 14^b^	[21, 52, 70]
	Forestry residues	6	–		−10	10 to 12^b^	[24, 52]
HTL (in situ)	Forestry residues	18	27^c^	[56]	18	–	
HTL (ex situ)	Forestry residues	21	–		21	–	
Pyrolysis (in situ)	Forestry residues	22	34^c^	[56]	22	–	
Pyrolysis (ex situ)	Forestry residues	41	–		37	–	
ATJ	Corn	54	–		71	–	
	Corn stover	35	–		22	–	
	Sugarcane	31	–		31	−27^d^	[26]
DSHC (increased blend level)	Sugarcane	76	–		79	55 to 100	[27]
DSHC (10% blend)	Sugarcane	47	–		49	–	

^a^Some conversion pathways could not be compared due to lack of reference studies. It should be noted that the literature entails a much wider feedstock and technology scope than employed in this study, including a wide range of LCAs of RJF production based on algae species, edible oil crops, and herbaceous crops [71, 72]

^b^Elgowainy et al. [24], Stratton et al. [21] and Stratton et al. [52] assume all electricity produced during FT synthesis is used internally

^c^Based on diesel production, not RJF. It is included in this comparison as it is used as a data source for our computations

^d^Relative to Staples et al. [26], this study uses lower yields and a higher electricity emission intensity

### Improving the GHG emission performance of RJF production

The second aim of this paper was to identify improvements inside and outside the RJF supply chain which lead to further GHG emission reductions. The GHG emission reduction performance of RJF may improve in the future by higher conversion yields, better agricultural practice and lower carbon intensity of utilities. At the same time, the emission intensity of fossil jet fuel will likely increase in the future as the trend towards the utilization of more heavy and sour (high sulfur) oil pursues [52, 73]. Moreover, relocation of RJF production can improve the GHG emission reduction performance significantly; particularly due to the relatively high emission intensity of the US electricity mix (see Table 3).

The production and use of hydrogen plays a particularly important role in current and future RJF production, as it is required in almost all pathways. Hence, sustainable hydrogen production technologies can have an important contribution towards reducing the emission intensity of RJF, especially when produced through electrolysis from renewable electricity. Furthermore, hydrogen consumption can sometimes be limited due to choice of feedstock, product slate, catalyst, organism or process conditions.

Deoxygenation remains inevitably important as oxygen is essentially the main impurity in biomass compared to RJF. In general, oxygen can be removed as water (using hydrodeoxygenation) and/or (biogenic) carbon dioxide (using decarboxylation, fermentation or gasification). Provided hydrogen can be produced sustainably, hydrodeoxygenation may be preferred from a climate change mitigation point of view as it increases conversion (carbon) yields and limits the emissions of biogenic carbon dioxide.^6^ On the other hand, pathways removing oxygen through carbon dioxide (particularly FT and fermentation pathways, but also hydrogen production from biomass gasification) yield high-purity point-source CO_2_ streams which can be captured against modest cost compared to lower-purity CO_2_ streams from power plants (fossil and bioenergy-based) [74–76]. Such bioenergy and carbon capture and storage (BECCS) options provide the opportunity to achieve negative emission performance for RJF and can contribute significantly to deep emission reductions on a global scale [77–79].

### Implications for a global meta-standard for RJF

The third aim of this study was to provide input to a global meta-standard for the calculation of the GHG emission performance of RJF. Whereas methodological differences can and should be smoothened in a global meta-standard for RJF to avoid competitive distortion or adverse sustainability effects, spatial differences are real and should ideally be addressed. Existing databases such as BioGrace, GREET, and GHGenius could be used as a starting point to determine regional default values (e.g., energy input and emission factors).

Co-product allocation is of particular importance for RJF production, as co-products are produced in almost all pathways (particularly fuel co-products in thermochemical pathways). The results of this study indicate that the choice for energy allocation or a hybrid method particularly affects pathways producing high amounts of (non-energy) co-products or co-products which effectively displace carbon intensive products (e.g., electricity in a US context).

Given the results and the trade-offs between different allocation methods (see “Methods to deal with co-products” section), we propose to employ energy allocation as a base in a global meta-standard, supplemented with economic allocation for specific systems. Energy allocation would likely lead to easier development and implementation, due to its universal character, indifference to the choice of main product and ability to capture the value of energy products. For non-energy co-products produced in specific systems, economic allocation was deemed appropriate as it is subject to fewer methodological and circumstantial choices than the displacement method.

Such framework necessitates a threshold co-product allocation ratio after which economic allocation is to be used and an index (or regional indices) on the basis of which the co-product allocation ratio should be determined, including a defined time span and sensible proxies for non-commoditized co-products. Moreover, it is important to be aware that this combination of allocation methods is sensitive to changes in co-product use (e.g. using naphtha as a chemical feedstock rather than using it for fuel production) or the product slate [e.g., produce more (non-energy) co-products at the expense of RJF yield] [21]. As some of the conversion pathways considered are flexible in product output (e.g. FT and HEFA), further research on the impact of product slate variability is encouraged.

### RJF as an emission mitigation instrument for aviation

The mitigation costs of RJF are high compared to other mitigation options for aviation. Combining techno-economic data from Jong et al. [6] with the results of this study yields minimum GHG emission mitigation costs of roughly 200 $/t CO_2eq_ abated, irrespective of co-product method (found for HTL at an oil price of 45 $/bbl). Although this figure is indicative and highly dependent on the oil price, these mitigation costs place RJF at the higher end of other biomass-based mitigation options [80].

Other mitigation options for aviation (e.g., carbon offsets or efficiency improvements in technology and operations) yield lower mitigation costs; most efficiency improvement measures come at zero or negative mitigation costs [79], while 85% of the global carbon offsets is currently priced at less than 10 US $/t CO_2_ [82]. Although carbon prices are expected to rise, it is unlikely that carbon prices will approach the mitigation costs for RJF before 2050 [83–85].

Nonetheless, the introduction of RJF is deemed an important part of the industry’s ambition to structurally reduce GHG emissions [4]. Hence, even though the Carbon Offsetting and Reduction Scheme for International Aviation (CORSIA) will raise the price of fossil fuel, it is most likely that further reduction of RJF production costs (through technological learning and maturation of biomass markets) and supplementary incentives are still required in order for airlines to prefer RJF adoption over buying emission credits to comply with the CORSIA scheme on the basis of cost. Given the substantial development efforts still required to get sufficient volumes of RJF on the market, the aviation sector cannot afford to rely solely on offsets and efficiency measures for the coming decade; it will need to continue to actively stimulate the development of RJF capacity in concurrence with the biofuel and biochemical sectors.

### Wider sustainability considerations of RJF production

The results of this analysis alone do not fully represent the climate change mitigation potential of RJF nor give a guarantee of the overall sustainability of RJF production. Firstly, this analysis does not include direct or indirect LUC emissions. Including LUC effects would likely lead to a stronger preference for residues. The magnitude of LUC emissions may have a positive or negative impact depending on the feedstock cultivation context (see “Land use change” section). Moreover, the importance of LUC effects is likely to increase with growing demand for RJF and other biomass-derived products [65].

Secondly, the GHG emission reduction as a result of using RJFs is not immediate. The timing of GHG emission savings (as captured in the GHG payback period) depends on the feedstock used and prior land use, since there generally exists a temporal imbalance (‘carbon debt’) between the time of emission and sequestration of the carbon. The GHG payback period is particularly long for feedstocks with long rotation periods and/or natural decay times, such as different types of woody biomass [86]. For the feedstocks investigated in this paper (residues, annual agricultural crops or short rotation crops), this effect is probably less relevant.

Thirdly, the system boundaries and functional unit employed in this analysis exclude the contribution to radiative forcing of other emission species than CO_2_, N_2_O and CH_4_. For example, emissions of water vapor, NO_*x*_, soot and sulfate aerosols, as well as contrails and contrail-induced cirrus formation caused by fuel combustion increase the radiative forcing by a factor 2–5 relative to the impact of CO_2_ emissions alone [87]. Although RJF has the potential to reduce some of these combustion emissions (particularly CO, NO_*x*_, PM_10_ and SO_*x*_) [24, 35], the positive impact of RJF on radiative forcing is likely to be lower than the percentage reduction in life-cycle GHG emissions suggests [32]. Furthermore, other emissions during the life-cycle (e.g., black carbon or primary organic carbon) or LUC-induced surface albedo effects may also significantly impact the net radiative forcing effect of biofuels [88–90].

Lastly, an assessment of the sustainability of RJF should also include other possible impacts on water use, land use, air quality, health effects, food security, and biodiversity, most of which are highly circumstantial and transcend the domain of RJF [9, 24, 91, 92].

## Conclusion

This study compares the well-to-wake (WtWa) GHG emission performance of various RJF conversion pathways and shows the impact of different co-product allocation procedures. Conversion pathways based on residues or lignocellulosic crops yield low WtWa GHG emissions, irrespective of allocation method. The FT pathway shows the highest GHG emission savings (86–104%) of the pathways considered, followed by HTL (77–80%), pyrolysis (54–75%), UCO-based HEFA (68%), and sugarcane- (71–75%) and corn stover-based ATJ (60–75%). The largest differences between energy allocation and the hybrid method (using the displacement method for non-fuel co-products) were found for conversion pathways producing high amounts of co-products or co-products which effectively displace carbon intensive products, such as FT, jatropha-based HEFA or corn-based ATJ. This study was framed in a particular spatiotemporal context; a comparison of RJF production across regions and timeframes using different assessment frameworks is recommended to determine the impact of methodological and actual differences on the GHG emission intensity of RJF production. Also, this assessment does not include emissions from land use change and could, thus, over- or underestimate the GHG emission performance in specific contexts.

The GHG emission performance of RJF can be enhanced by using more sustainable sources of electricity and hydrogen (e.g., biomass or renewable electricity), improving agricultural practices and advancing RJF technologies. Also, some pathways provide the opportunity to be combined with carbon capture and storage, potentially yielding negative emissions at relatively modest cost compared to other options for carbon capture and storage. Future research should evaluate the potential of these improvement options, preferably from a broader energy systems perspective.

The inclusion of RJF in a global carbon offsetting scheme requires a harmonized methodology to assess the GHG emission performance of different RJFs. We recommend using energy allocation as a base, supplemented with economic allocation for systems yielding high shares of non-energy co-products. This combination of allocation methods leverages the universal character of energy allocation and the ability of economic allocation to properly value non-energy co-products. The allocation methodology is only one of the aspects of a global meta-standard; broad cooperation is required to develop a robust framework which needs to be flexible to account for spatial diversity yet standardized to avoid competitive distortion or adverse sustainability effects.

## Additional files

**Additional file 1.** Energy allocation and displacement method.

**Additional file 2.** Input data.

**Additional file 3.** Sensitivity analysis yield ranges.

## Footnotes

1 To leverage the experience with biofuels in the road transport sector and avoid adverse sustainability effects, the Sustainable Aviation Fuel Users Group (SAFUG—a group of airlines representing approximately one-third of global jet fuel use), has committed to using fuels which do not compete with food supplies, significantly reduce well-to-wake GHG emissions, and have a low risk of indirect land use change (LUC) [93, 94].

2 For example, a fundamental difference between the renewable energy directive (RED) and renewable fuel standard (RFS) is the amortization period; whereas the RED specifies 20 years, the RFS uses 30 years.

3 HEFA, FT, DSHC and ATJ (from butanol) are certified for use in commercial aviation by the American Society of Testing and Materials (ASTM). The other pathways are currently in the certification process.

4 As combustion emissions were excluded in the source used [53], the CO_2_ emissions from the combustion of conventional jet fuel as reported in Stratton et al. [52] (73.2 g CO_2_/MJ) were added.

5 Commercial Aviation Alternative Fuels Initiative (CAAFI’s) Fuel Readiness Level (FRL) methodology is based on NASA’s Technology Readiness Level (TRL) scheme. The FRL scale allows users to track the progress of a RJF in terms of research, certification, and demonstration [95].

6 Life-cycle GHG emission assessments generally assume biogenic carbon dioxide emissions to be fully offset by carbon sequestration during feedstock growth. Nonetheless, such emissions do contribute to radiative forcing until they are sequestered and as such influence the timing of GHG emission savings (as captured in the carbon payback time).
