# Supplementary material for: Life-cycle analysis of greenhouse gas emissions from renewable jet fuel production
Source: Biotechnol Biofuels. 2017 Mar 14;10:64. doi: 10.1186/s13068-017-0739-7 (PMC5348797; doi:10.1186/s13068-017-0739-7)
Supplement: Supplementary file 1 — Additional file 1. Energy allocation and displacement method. [file 13068_2017_739_MOESM1_ESM.pdf]

3 Energy allocation and displacement method

For a hypothetical system with one main product and one co-product the GHG emission intensity of the main product can be calculated as follows:

$$Energy\ allocation: \varepsilon_{main\ product,EA} = \varepsilon_{system} \quad (1)$$

$$Displacement: \ \varepsilon_{main\ product,DM} = \frac{\varepsilon_{system} - (1-\eta_p) * r_D * \varepsilon_{displaced\ product}}{\eta_p} \quad (2)$$

In which ε is the specific GHG emission intensity for the total system (including the product and co-products) and the displaced product. The product share (η_p), co-product allocation ratio (r_A) and displacement ratio (r_D) are defined here as:

$$\eta_p = \frac{1}{1+r_A} = \frac{E_p}{E_p + E_{cp}} \ in\ which\ r_A \equiv \frac{E_{cp}}{E_p} \quad (3)$$

$$r_D \equiv \frac{E_{cp}}{E_{dp}} \quad (4)$$

In which E is the energy content of the product (p), co-product (cp) and displaced product (dp). The parameters η_p and r_D can be defined in terms of units (e.g. kg/kg, MJ/MJ). To avoid conversion, we use MJ/MJ in this example. It should be noted that η_p is inversely proportional to r_A. Furthermore, equation 1 and 2 show that while energy allocation yields strictly positive emission intensities (excluding the possibility that the total system is carbon negative due to the application of carbon capture and storage), the displacement method may give negative emission intensities.

The difference (Δ) between the GHG emission intensity of the main product using energy allocation and the displacement method is expressed as:

$$\begin{aligned}\Delta = \varepsilon_{EA} - \varepsilon_{DM} = & \left(\frac{1}{\eta_p} - 1\right) * \left(r_D * \varepsilon_{displaced\ product} - \varepsilon_{system}\right) \\ = & \ r_A * \left(r_D * \varepsilon_{displaced\ product} - \varepsilon_{system}\right) \ (5)\end{aligned}$$

The sign of the difference is determined by the term $r_D * \varepsilon_{displaced\ product} - \varepsilon_{system}$. Hence, energy allocation yields higher GHG emissions when:

$$r_D * \varepsilon_{displaced\ product} > \varepsilon_{system} \ (6)$$

The displacement method yields higher GHG emissions for systems in which:

$$r_D * \varepsilon_{displaced\ product} < \varepsilon_{system}\ (7)$$

As an illustration we take a system in which the co-product is green electricity. As electricity is often a direct substitute for grid electricity r_d is likely equal to 1. As such, inequalities 6 and 7 reduce to a comparison between the emission intensity of the displaced product (grid electricity) and the energy intensity of the system. For systems having lower specific emission intensity than grid electricity, the displacement method will always yield a lower GHG emission intensity for the main product. For systems having higher emission intensity than grid electricity, the energy allocation method will always yield lower results than the displacement method.

The size of Δ grows with higher co-product allocation ratios and increasing divergence between the terms $r_D * \varepsilon_{displaced\ product}$ and ε_{system}. In other words, larger differences between the results of both methods may be expected for systems producing high amounts of co-products. Furthermore, for systems which are much less emission intensive than the system producing the displaced product (i.e. $\varepsilon_{displaced\ product} \gg \varepsilon_{system}$) larger differences between both methods occur for systems producing co-products that effectively displace emission intensive products (i.e. high r_D and $\varepsilon_{displaced\ product}$).

For systems with more co-products the general dynamics still hold, i.e. higher allocation ratios will lead to higher differences. Calculation of the sign of the difference requires, however, more parameters than postulated for the example system above.
