# Supplementary material for: Life-cycle analysis of greenhouse gas emissions from renewable jet fuel production
Source: Biotechnol Biofuels. 2017 Mar 14;10:64. doi: 10.1186/s13068-017-0739-7 (PMC5348797; doi:10.1186/s13068-017-0739-7)
Supplement: Supplementary file 2 — Additional file 2. Input data. [file 13068_2017_739_MOESM2_ESM.docx]

## Input data

All feedstock cultivation and conversion was considered to occur in the US, except sugar cane-based ATJ and DSHC (Brazil). For sugarcane-based conversion pathways all cultivation and conversion inputs correspond to a Brazilian context. Consequent downstream transport of RJF from Brazil to the US was accounted for (see section 1.5).

### Feedstock cultivation

The inputs for feedstock cultivation are displayed in Table 1. The emission factors corresponding to the inputs were obtained from GREET. Emissions for the production of residues (corn stover, forestry residues) or food by-products (used cooking oil) were considered from the time of collection. It was not chosen to allocate a portion of the cultivation emissions to the residue or by-product to avoid additional assumptions on input data and allocation methodology. Fertilizer inputs for corn stover were based on the required supplementary fertilizer to replenish soil nutrients after corn stover harvesting; no fertilizer for the production of forest residue was assumed.[1] Used cooking oil was considered a recycled product, hence emissions from used cooking oil production were excluded.[2–4] Emissions from residue collection were included.

Besides emissions from fertilizer production and application, N_2_O emissions are also caused by direct and indirect conversion of N fertilizers in the soil [5]. Direct emissions result from nitrification and denitrification cycles. Indirect emissions are caused by nitrate leaching and volatilization of nitrates [6]. The conversion rate to calculate the N_2_O emissions per unit of N fertilizer applied is highly uncertain as it depends on local factors such as soil and climate conditions, farm practices, N fertilizer type, and application rate.[6–9]

The reference studies used in this analysis utilize different conversion rates to calculate the N_2_O emissions from N fertilizer. For the majority of the feedstocks (and poplar, willow, forest residue, corn stover, corn and sugarcane) the conversion rates as specified in GREET were used (1.525% for poplar, willow, forestry residues, corn stover, and corn, and 1.22% for Brazilian sugarcane) [6]. These factors were determined from an extensive literature survey. N_2_O emissions from jatropha and camelina cultivation were calculated using the IPCC Tier 1 method^[[1]](#footnote-1)^, reporting conversion rates of 1.325% for N fertilizers and 1.225% nitrogen from crop residues.[7,9]

The conversion rates as specified in the reference study were also adopted in this study. To account for the uncertainties associated with N_2_O soil emissions, the impact of fertilizer use was analyzed in the sensitivity analysis.

Table 1: Input values for feedstock cultivation

|  | | **Fertilizer** | | | | | | **Utilities** | | | | | **Other inputs** | | **Geography** | **Source** |
| --- | --- | --- | --- | --- | --- | --- | --- | --- | --- | --- | --- | --- | --- | --- | --- | --- |
|  |  | K2O | P2O5 | N | CaCO3 | Herbicides | Insecticides | Diesel | Natural gas | Electricity | Gasoline | LPG |  |  |  |  |
|  | Product | g/kg product | g/kg product | g/kg product | g/kg product |  | - | MJ/kg product | MJ/kg product | MJ/kg product | MJ/kg product | MJ/kg product |  | g/kg product |  |  |
| Jatropha | Jatropha seeds | 40.20 | 14.00 | 36.60 | - | - | - | 1.50 | - | - | - | - |  |  | US | [10] |
| Camelina | Camelina Seeds | 11.60 | 17.40 | 43.00 | - | - | - | 1.18 | - | - | - | - |  |  | US | [10] |
| UCO | UCO | - | - | - | - | - | - | - | - | - | - | - |  |  | US | [11,2] |
| Poplar | Poplar | 2.02 | 1.01 | 3.02 | 2.38 | 0.15 | - | 0.25 | - | - | - | - |  |  | US | [10] |
| Willow | Willow | - | - | 2.85 | - | 0.03 | - | 0.18 | 0.00 | 0.00 | - | - |  |  | US | [10] |
| Corn stover | Corn stover | 15.04 | 8.77 | 8.77 | - | - | - | 0.26 | - | - | - | - |  |  | US | [10] |
| Forestry residues | Forestry residues | - | - | - | - | - | - | 0.14 | - | - | - | - |  |  | US | [10] |
| Sugarcane | Sugarcane | 1.00 | 0.30 | 0.80 | 5.20 | 0.05 | 2.50 | 0.04 | 0.02 | 0.01 | 0.01 | 0.02 | Sugarcane straw^i^ | 17 | Brazil | [10] |
| Corn | Corn | 5.39 | 5.20 | 15.09 | 40.98 | 0.28 | 2.36 | 0.18 | 0.05 | 0.02 | 0.05 | 0.06 |  |  | US | [10] |

1. The straw is assumed to be burnt on the field

### Feedstock pre-processing

Table 2 shows the assumptions for feedstock pre-processing steps.

Table 2: Input values for feedstock pre-processing

| **Feedstock** | **Process** | **Yield** |  | **Other inputs** | | | **Co-product** | | | **Source** |
| --- | --- | --- | --- | --- | --- | --- | --- | --- | --- | --- |
| Jatropha | Oil extraction | 74.36 | g seed/MJ oil | Natural gas | 0.049 | MJ/MJ oil | Electricity | 0.34 | MJ/MJ oil | [9,10] |
|  |  |  |  | Hexane | 0.0047 | MJ/MJ oil |  |  |  |  |
| Camelina | Oil extraction | 74.36 | g seed/MJ oil | Natural gas | 0.031 | MJ/MJ oil | Camelina meal | 47.79 | g/MJ oil | [7,10] |
|  |  |  |  | Hexane | 0.0027 | MJ/MJ oil |  |  |  |  |
|  |  |  |  | Electricity | 0.0023 | MJ/MJ oil |  |  |  |  |
|  |  |  |  | Diesel | 0.017 | MJ/MJ oil |  |  |  |  |
| UCO | Rendering | 1.66 | kg/kg yellow grease | Natural gas | 0.039 | MJ/MJ oil |  |  |  | [11,2] |
|  |  |  |  | Electricity | 0.0040 | MJ/MJ oil |  |  |  |  |

### Upstream transport

Table 3 shows the assumptions used for feedstock transportation. Several transportation modes are used in GREET.net. The pathways in the scope of this research include heavy-duty trucks, train and barges. Dunn et al.[12] reports assumptions for the energy intensity and emissions parameter for transportation used in GREET. The energy intensity for goods transport by rail is based on data reported to the Surface Transportation Board of the United States Department of Transportation (DOT). Emission factors are based on values from EPA. The energy intensity and emissions factors for Heavy-Duty trucks are based on EPA’s Motor Vehicle Emission Simulator (MOVES) model. GREET includes a Medium Heavy-Duty Truck and a Heavy Heavy-Duty truck. The energy intensity and emission values for Barge transport is based on research by the Bureau of Transportation statistics and the Department of Energy. Transportation distances and mode shares are based on the Freight Analysis Framework (FAF) model of the DOT. The payloads and transport parameters can be found in GREET [10].

Table 3: Upstream transport assumptions

| **Transport** |  |  |  |  |  |  |
| --- | --- | --- | --- | --- | --- | --- |
| Feedstock | Transport stage | Transport mode | Distance | Share | Geography | Source |
|  |  |  | km |  |  |  |
| Jatropha | From field to stacks | Medium Heavy-Duty Truck | 16 | 100% | US | [10] |
|  | From stacks to extraction plant | Heavy Heavy-Duty Truck | 64 | 100% | US |  |
|  | From extraction plant to conversion plant | Barge | 837 | 40% | US |  |
|  |  | Rail | 1127 | 20% | US |  |
|  |  | Heavy Heavy-Duty Truck | 129 | 40% | US |  |
| Camelina | From field to stacks | Medium Heavy-Duty Truck | 16 | 100% | US | [10] |
|  | From stacks to extraction plant | Heavy Heavy-Duty Truck | 64 | 100% | US |  |
|  | From extraction plant to conversion plant | Rail | 1127 | 33% | US |  |
|  |  | Heavy Heavy-Duty Truck | 129 | 67% | US |  |
| UCO | From collection location to rendering plant | Heavy-Heavy Duty truck | 156 | 100% | US | [11] |
|  | From rendering plant to conversion facility | Heavy Heavy-Duty Truck | 80 | 100% | US | [10] |
| Poplar | From field to conversion plant | Heavy Heavy-Duty Truck | 80 | 100% | US | [10] |
| Willow | From field to conversion plant | Heavy Heavy-Duty Truck | 80 | 100% | US | [10] |
| Corn stover | From field to conversion plant | Heavy Heavy-Duty Truck | 153 | 100% | US | [10] |
| Forestry residues | From field to conversion plant | Heavy Heavy-Duty Truck | 144 | 100% | US | [10] |
| Sugar cane | From field to conversion plant | Heavy Heavy-duty truck | 19.31 | 100% | Brazil | [10] |
| Corn | From corn field to stack | Heavy Heavy-duty truck | 64 | 100% | US | [10] |
|  | From stacks to ethanol plant | Medium Heavy-Duty Truck | 16 | 100% | US |  |

### Conversion to RJF

The table below provides supplementary data not tabulated in Table 2 and Table 3 in the main text. It should be noted that the level of detail varies among the reference studies. For example, whereas the catalysts are included for ATJ, they are not included for FT. Similarly, enzyme and yeast use for sugarcane ethanol production is not included in GREET as the reference study of Wang et al.[6] did not have date available and assumed that their effect on sugarcane WTWa GHG emissions are small, as is the case for corn ethanol. Based on similar reasoning we are confident that this study has included the most important inputs from a GHG point of view.

| **Input** | **Unit** | **Value** | | | **Source** |
| --- | --- | --- | --- | --- | --- |
| **DSHC** |  |  | | |  |
| Yeast | g/MJ Jet | 1.41E-06 | | | [13] |
| Sodium chloride | g/MJ Jet | 9.95 | | |  |
| Sodium hydroxide | g/MJ Jet | 0.23 | | |  |
| Ammonium hydroxide | g/MJ Jet | 2.59 | | |  |
| Diammonium phosphate | g/MJ Jet | 0.63 | | |  |
| Glucose | g/MJ Jet | 22.49 | | |  |
| Sulfuric acid | g/MJ Jet | 11.7 | | |  |
|  |  |  | | |  |
| **ATJ** |  |  | | |  |
| Catalyst for hydrotreating | g/MJ Jet | 0.064 | | | [14] |
| Catalyst for oligomerization | g/MJ Jet | 0.043 | | |  |
|  |  |  | | |  |
| **Corn stover to ethanol** |  |  | | |  |
| Corn stover | kg/MJ ethanol | 0.13 | | | [10] |
| Sulfuric acid | g/MJ ethanol | 3.10 | | |  |
| Ammonia | g/MJ ethanol | 1.77 | | |  |
| Yeast | g/MJ ethanol | 0.35 | | |  |
| Cellulase | g/MJ ethanol | 1.41 | | |  |
| Diesel | MJ/MJ ethanol | 0.00 | | |  |
|  |  |  | | |  |
| **Sugarcane to ethanol^i^** |  |  | | |  |
| Sugarcane | g/MJ ethanol | 579.89 | | | [10] |
| Residual oil | g/MJ ethanol | 3.93E-03 | | |  |
|  |  |  | | |  |
| **Corn to ethanol^ii^** |  | **1** | **2** | **3** |  |
| Alpha Amylase | g/MJ ethanol | 0.03 | 0.03 | 0.03 | [10] |
| Gluco Amylase | g/MJ ethanol | 0.07 | 0.07 | 0.07 |  |
| Yeast | g/MJ ethanol | 0.03 | 0.03 | 0.04 |  |
| Sulfuric Acid | g/MJ ethanol | 0.22 | 0.22 | 0.23 |  |
| Ammonia | g/MJ ethanol | 0.22 | 0.22 | 0.23 |  |
| Sodium hydroxide | g/MJ ethanol | 0.27 | 0.27 | 0.29 |  |
| Calcium oxide | g/MJ ethanol | 0.13 | 0.35 | 0.14 |  |
| Natural Gas | MJ/MJ ethanol | 0.29 | 0.29 | 0.45 |  |
| Electricity | MJ/MJ ethanol | 0.03 | 0.034 | - |  |
| Coal | MJ/MJ ethanol | 2.00E-03 | 0.03 | 0.17 |  |

1. Sugarcane straw and sugarcane bagasse is used for internal heat and power.
2. GREET uses a weighted average of three different corn ethanol technologies. Dry mill ethanol production without corn oil extraction (1), dry mill ethanol production with corn oil extraction (2), and wet mill ethanol production (2) respectively produce 18.23%, 72.91% and 8.87% of the total produced ethanol.

### Downstream distribution

Downstream distribution includes the transportation of the RJF to a blending terminal, blending operations, transportation to the airport tank farm, storage and distribution in the airport hydrant system. Input data is listed in Table 4 and Table 5. Electricity use for storage and blending was obtained from BioGrace [4]. Similar to BioGrace, variance in energy use as a result of different blend walls for each RJF type was not taken into account.

Table 4: Energy use for blending and storage

| **Supply chain component** | **Value** | **Unit** | **Source** |
| --- | --- | --- | --- |
| Blending (filling station) | 0.0034 | MJ electricity/MJ RJF | [4] |
| Storage (depot) | 0.00084 | MJ electricity/MJ RJF | [4] |

Table 5: Transport distance downstream distribution

| **Transport** |  |  |  |  |  |  |
| --- | --- | --- | --- | --- | --- | --- |
| Product | Transport stage | Transport mode | Distance | Share | Geography | Source |
|  |  |  | km |  |  |  |
| RJF | Conversion plant to bulk terminal | Barge | 837 | 8% | US | [10] |
|  |  | Rail | 1287 | 29% | US | [10] |
|  |  | Heavy Heavy-Duty Truck | 80 | 63% | US | [10] |
| RJF | Blending terminal to airport farm | Heavy Heavy-Duty Truck | 48 | 100% | US | [10] |
| RJF^i^ | Conversion plant to bulk terminal | Heavy Heavy-Duty Truck | 692 | 100% | Brazil | [10] |
| RJF^i^ | Bulk terminal Brazil to US terminal | Small Ocean Tanker | 11935 | 100% | Brazil-US | [10] |
| RJF^i^ | US terminal to blending terminal | Heavy Heavy-Duty Truck | 48 | 100% | US | [10] |

1. Only applicable for sugarcane-based pathways

## References

1. Han J, Elgowainy A, Palou-Rivera I, Dunn JB, Wang MQ. Well-to-Wheels Analysis of Fast Pyrolysis Pathways with GREET. 2011.

2. Seber G, Malina R, Pearlson MN, Olcay H, Hileman JI, Barrett SRH. Environmental and economic assessment of producing hydroprocessed jet and diesel fuel from waste oils and tallow. Biomass and Bioenergy. 2014;67:108–18.

3. Talens Peiró L, Lombardi L, Villalba Méndez G, Gabarrell i Durany X. Life cycle assessment (LCA) and exergetic life cycle assessment (ELCA) of the production of biodiesel from used cooking oil (UCO). Energy. 2010;35:889–93.

4. BioGrace. BioGrace - Excel based biofuel GHG calculations. Version 4d. http://www.biograce.net/home2015 Accessed 2016 Jul 9.

5. Elgowainy A, Han J, Wang M, Carter N, Stratton R, Hileman J. Life-Cycle Analysis of Alternative Aviation Fuels in GREET. 2012.

6. Wang M, Han J, Dunn JB, Cai H, Elgowainy A. Well-to-wheels energy use and greenhouse gas emissions of ethanol from corn, sugarcane and cellulosic biomass for US use. Environ. Res. Lett. 2012;7:45905.

7. Shonnard DR, Williams L, Kalnes TN. Camelina-Derived Jet Fuel and Diesel: Sustainable Advanced Biofuels. 2010;29:383–92.

8. Edwards R, Larive J-F, Rickeard D, Weindorf W. Well-to-Wheels analysis of future automotive fuels and powertrains in the European context WELL-TO-TANK (WTT) Report. Version 4. 2014.

9. Stratton RW, Wong HM, Hileman JI. Life Cycle Greenhouse Gas Emissions from Alternative Jet Fuels. 2010.

10. Argonne National Laboratory. Greenhouse Gases, Regulated Emissions, and Energy Use in Transportation (GREET) GREET.net Computer Model. https://greet.es.anl.gov/index.php?content=greetdotnet2015 Accessed 2016 Feb 2.

11. López DE, Mullins JC, Bruce DA. Energy life cycle assessment for the production of biodiesel from rendered lipids in the United States. Ind. Eng. Chem. Res. 2010;49:2419–32.

12. Dunn JB, Elgowainy A, Vyas A, Lu P, Han J, Wang M, et al. Update to Transportation Parameters in GREET. 2013.

13. Klein-Marcuschamer D, Turner C, Allen M, Grey P, Dietzgen R, Gresshoff P, et al. Technoeconomic analysis of renewable aviation fuel from microalgae, Pongamia pinnata, and sugarcane. Biofuels. Bioprod., Bioref. 2013;7:416–28.

14. Argonne National Laboratory. Greenhouse Gases, Regulated Emissions, and Energy Use in Transportation (GREET) GREET_1_2015 Excel model. https://greet.es.anl.gov/greet_1_series2015 Accessed 2016 Feb 2.

15. Intergovernmental Panel on Climate Change. N2O Emissions From Managed Soils, and CO2 Emissions From Lime and Urea application. 2006 IPCC Guidel. Natl. Greenh. Gas Invent. Vol. 4 Agric. For. Other L. Use. 2006. p. 11.1-11.54.

1. More information on the IPCC Tier 1-3 methods can be found in [15]. [↑](#footnote-ref-1)
