# Supplementary material for: Life-cycle analysis of greenhouse gas emissions from renewable jet fuel production
Source: Biotechnol Biofuels. 2017 Mar 14;10:64. doi: 10.1186/s13068-017-0739-7 (PMC5348797; doi:10.1186/s13068-017-0739-7)
Supplement: Supplementary file 3 — Additional file 3. Sensitivity analysis yield ranges. [file 13068_2017_739_MOESM3_ESM.pdf]

2 Sensitivity analysis yield ranges[1]

		**Base**	**Pessimistic**	**Optimistic**
		kg fuel product/kg feed		
HEFA	UCO	0.88	0.75	0.89
	Camelina oil	0.88	0.75	0.89
	Jatropha oil	0.88	0.75	0.89
FT	Poplar	0.19	0.13	0.22
	Willow	0.19	0.13	0.22
	Corn stover	0.18	0.13	0.22
	Forestry residues	0.21	0.13	0.22
HTL in-situ	Forestry residues	0.27	0.17	0.36
HTL ex-situ	Forestry residues	0.27	0.17	0.36
Pyrolysis in-situ	Forestry residues	0.24	0.26	0.36
Pyrolysis ex-situ	Forestry residues	0.24	0.26	0.36
ATJ	Ethanol	0.54	0.53	0.58
DSHC increased blend wall	Sugar[i]	0.18	0.15	0.30
DSHC 10%	Sugar[i]	0.18	0.15	0.30

i. Sugar to farnasene yield`

References

1. de Jong S, Hoefnagels R, Faaij A, Slade R, Mawhood R, Junginger M. The feasibility of short-term production strategies for renewable jet fuels – a comprehensive techno-economic comparison. Biofuel, Bioprod. Bioref. 2015;9:778–800.
